# STK39 is a novel kinase contributing to the progression of hepatocellular carcinoma by the PLK1/ERK signaling pathway

**DOI:** 10.7150/thno.48112

**Published:** 2021-01-01

**Authors:** Chengfei Zhang, Xiaoming Wang, Dan Fang, Ping Xu, Xiao Mo, Chao Hu, Alaa Abdelatty, Mei Wang, Haojun Xu, Qi Sun, Guoren Zhou, Junjun She, Jinglin Xia, Kam Man Hui, Hongping Xia

**Affiliations:** 1 Department of Pathology, School of Basic Medical Sciences & Sir Run Run Hospital & State Key Laboratory of Reproductive Medicine & Key Laboratory of Antibody Technique of National Health Commission, Nanjing Medical University, Nanjing 211166, Jiangsu, China.; 2 Department of Hepatobiliary Surgery, The First Affiliated Hospital of Wannan Medical College, Wuhu 241001, Anhui, China.; 3 Jiangsu Cancer Hospital, The Affiliated Cancer Hospital of Nanjing Medical University, Jiangsu Institute of Cancer Research, Nanjing 210009, Jiangsu, China.; 4 Department of High Talent & General Surgery & Med-X Institute, The First Affiliated Hospital of Xi'an Jiao Tong University, Xi'an, 710061, Shaanxi, China.; 5 The First Affiliated Hospital of Wenzhou Medical University, Wenzhou 325000, Zhejiang, China.; 6 Laboratory of Cancer Genomics, National Cancer Centre Singapore & Cancer and Stem Cell Biology Program, Duke-NUS Medical School, Singapore.

**Keywords:** STK39, PLK1, ERK, HCC, proliferation

## Abstract

**Rationale:** Protein kinases are critical therapeutic targets for curing hepatocellular carcinoma (HCC). As a serine/threonine kinase, the potential roles of serine/threonine kinase 39 (STK39) in HCC remain to be explored.

**Methods:** The expression of STK39 was examined by RT-qPCR, western blotting and immunohistochemistry. Cell proliferation and apoptosis were detected by CCK8 and TUNEL kit. Cell migration and invasion assays were performed using a transwell system with or without Matrigel. RNA-seq, mass spectrometry and luciferase reporter assays were used to identify STK39 binding proteins.

**Results:** Here, we firstly report that STK39 was highly overexpressed in clinical HCC tissues compared with adjacent tissues, high expression of STK39 was induced by transcription factor SP1 and correlated with poor patient survival. Gain and loss of function assays revealed that overexpression of STK39 promoted HCC cell proliferation, migration and invasion. In contrast, the depletion of STK39 attenuated the growth and metastasis of HCC cells. Moreover, knockdown of STK39 induced the HCC cell cycle arrested in the G2/M phase and promoted apoptosis. In mechanistic studies, RNA-seq revealed that STK39 positively regulated the ERK signaling pathway. Mass spectrometry identified that STK39 bound to PLK1 and STK39 promoted HCC progression and activated ERK signaling pathway dependent on PLK1.

**Conclusions:** Thus, our study uncovers a novel role of STK39/PLK1/ERK signaling axis in the progress of HCC and suggests STK39 as an indicator for prognosis and a potential drug target of HCC.

## Introduction

As the most common type of primary liver cancer, hepatocellular carcinoma (HCC) is characterized by high malignancy, recurrence and metastasis rates. HCC is the second most common leading cause of cancer-related deaths worldwide 1-3. Many factors contribute to HCC development, including hepatitis B virus (HBV) and hepatitis C virus (HCV) infection, long-term alcohol consumption, aflatoxin B1 contaminated food and diabetes 4, 5. Despite knowing the risk factors of HCC, the detailed molecular mechanism underlying HCC largely remains to be elucidated 6. According to Global Cancer Statistics in 2018, the incidence rate of HCC is approximately equal to the mortality rate, stating that the clinical treatment effect of HCC is extremely poor 7. Currently, the treatments of HCC mainly include surgical resection, chemotherapy, molecular targeted therapy and immunotherapy 8, 9. Whereas, due to the insidious and aggressive features, most HCC patients are diagnosed at advanced disease stages or distant metastasis. In the patients who undergo surgical resection, the recurrence at five years is as high as 70% 10. Since 2007, oral multi-kinase inhibitors such as sorafenib, regorafenib, lenvatinib and cabozantinib have been gradually approved for HCC treatment. However, these drugs only prolong patients' life by a few months 11. Immune checkpoint inhibitors such as anti-PD-1 agent nivolumab showed a significant effect on some HCC patients by enhancing T cell activation, but its high non-response rate and potential risks still need to be addressed 12-14. Thus, a better understanding of the molecular mechanism underlying HCC and finding the new target for HCC is urgent.

Mitogen-activated protein kinase (MAPK)/extracellular signal-regulated kinase (ERK), phosphoinositide 3-kinase (PI3K)/protein-kinase B (AKT) and Wnt/β-catenin signaling pathways are crucial for the development of HCC. These oncogenic signaling pathways can be activated by multiple oncogenic drivers during hepatocarcinogenesis 15, 16. The activation of the MAPK/ERK signaling pathway has occurred in 50%-100% of HCC tumors. Overexpression or activation of this pathway contributes to cell proliferation, differentiation, apoptosis, cell cycle, metastasis and drug resistance in a variety of tumors 17-19. Constitutive activation of the PI3K/AKT signaling has been believed to be a significant determinant for cell growth and survival in a variety of cancers, which can be over-activated by tyrosine kinases, especially insulin-like growth factor receptor (IGFR) and epidermal growth factor receptor (EGFR) 16, 20. It has been reported that β-catenin is mutated in 17% and overexpressed in 50-70% of HCC tumors. Accumulation of β-catenin promotes the initiation, growth, survival, migration differentiation and apoptosis of HCC 16, 21. Improved knowledge of these signaling pathways helped us to identify several possible therapeutic targets for HCC. However, a broader understanding of how these signaling cascades are controlled by other oncogenic drivers remains elusive.

Polo-like kinase 1 (PLK1) is a serine/threonine kinase and consists of a conserved N-terminal kinase catalytic domain and a C-terminal polo-box domain (PBD) 22. As a key regulator of cell division, PLK1 has been reported as overexpression in a wide range of human tumors 23-25. Many studies have reported PLK1 as a critical driver in HCC and overexpressed PLK1 promotes cell growth during hepatocarcinogenesis, indicating that PLK1 is a promising therapeutic target for HCC 3, 26-28.

Serine/threonine kinase 39 (STK39) is a proline- and alanine-rich Ste20-related kinase (also named as SPAK), which is composed of a short N-terminal proline and alanine repeats (PAPA box), a kinase catalytic domain and a C-terminal regulatory domain 29, 30. In mammalians, STK39 plays an important role in ion homeostasis by regulating the cation chloride cotransporters' activities, which is crucial for the modulation of renal salt transport and blood pressure 31, 32. Recent studies have shown that STK39 expression is associated with tumor malignancy in some cancers 33-35. However, the molecular role and regulatory mechanism of STK39 in HCC remains unknown.

In the current study, we found that STK39 was upregulated in HCC and correlated with poor patient survival. Enforced expression of STK39 promoted growth and metastasis of HCC. In addition, we found that STK39 interacted with PLK1 and, thereby, led to the activation of the MAPK/ERK signaling pathway. Taken together, our data demonstrate that STK39 promotes the progression of HCC through the activation of the PLK1/ERK signaling pathway and, as such, STK39 may be used as a novel therapeutic target for HCC.

## Materials and methods

Additional methods are described in the Supporting Materials and Methods online.

### Generation of STK39-knockout cell line

To generate an STK39-knockout HCC cell line, the CRISPR/Cas9 system was used. Briefly, LentiCRISPRv2 plasmid containing the STK39-knockout target sequence 5ʹ- CGGCGGCACAGGCTGTCGGC-3ʹ was transduced into HuH7 cells. After two days of infection, puromycin-resistant single clones were selected, and the STK39-knockout clones were identified by western blotting with an anti-STK39 antibody.

### RNA-sequence

STK39-knockdown and control HuH7 cells were lysed in Trizol. The global gene expression profiles were examined by RNA sequencing in Novogene (Beijing, China). Biological process analyses were carried out with Enrichr tools.

### Immunoblot analysis

Cells were lysed in RIPA buffer (50 mM Tris, pH 7.4, 150 mM NaCl, 2 mM EDTA, 0.5% Nonidet P-40) containing protease and phosphatase inhibitors, and the protein concentration was measured using a BCA Protein Assay Kit (Beyotime). Cell lysates were separated by 10% SDS-PAGE, transferred to nitrocellulose membranes and blocked with 5% BSA. After hybridization with primary antibodies and appropriate secondary antibodies, protein bands were visualized with the chemiluminescence imaging system (Beijing Sage Creation). The density of protein bands was assessed by Image J software, and the protein levels were normalized to GAPDH.

### Immunoprecipitation

Cells were lysed in lysis buffer (50 mM Tris, pH 7.4, 150 mM NaCl, 2 mM EDTA, 0.5% Nonidet P-40, 10% glycerol, EDTA-free protease inhibitor cocktail) and then clarified by protein G agarose. Proteins were immunoprecipitated from the pre-cleared cell lysates with Flag, HA antibodies or control IgG at 4 °C overnight, and then pulled down by protein G agarose and assessed by immunoblotting.

### Xenograft tumor model

WT and STK39-knockdown or -knockout HuH7 cells (2×10^6^) were injected subcutaneously at bilateral sites in BALB/c nude mice (male, 4-6 weeks old). Tumor size was measured weekly. Tumor volume was measured with a caliper and calculated as length × width × width/2. Five weeks later, mice were euthanized and xenografted tumor tissues were dissected. Then, the weight of tumors was measured, and tumor tissues were submitted to IHC analysis.

### Mass spectrometry

HCCLM3 cells were infected with Flag-STK39 lentivirus for 48 h, and then the cells were treated with 2 μg/mL of puromycin for about one week. The HCCLM3-Flag-STK39 cells were collected and resuspended in lysis buffer (50 mM Tris, pH 7.4, 150 mM NaCl, 2 mM EDTA, 0.5% Nonidet P-40, 10% glycerol, EDTA-free protease inhibitor cocktail). Extracted proteins were immunoprecipitated with anti-Flag antibody or control IgG and separated by SDS-PAGE. After being visualized with coomassie brilliant blue staining, bands corresponding to control and Flag-STK39 were cut into small slices and digested with trypsin. The peptides were then desalinized and analyzed by mass spectrometry (Hangzhou Lc-Bio Technologies). The spectral data were analyzed in Proteome Discoverer, and protein peptides found in the anti-Flag sample more than 2-fold that of control sample were considered as potential binding partners of STK39. The complete original MS data were provided as separate excel files.

### Statistical analysis

All data were analyzed using the GraphPad Prism software and presented as the mean ± SEM. The Student's t-test (two-tailed) was used for comparison between groups. *P-*value <0.05 was considered statistically significant.

## Results

### STK39 is significantly upregulated in HCC and correlated with a poor outcome

**Figure 1:**
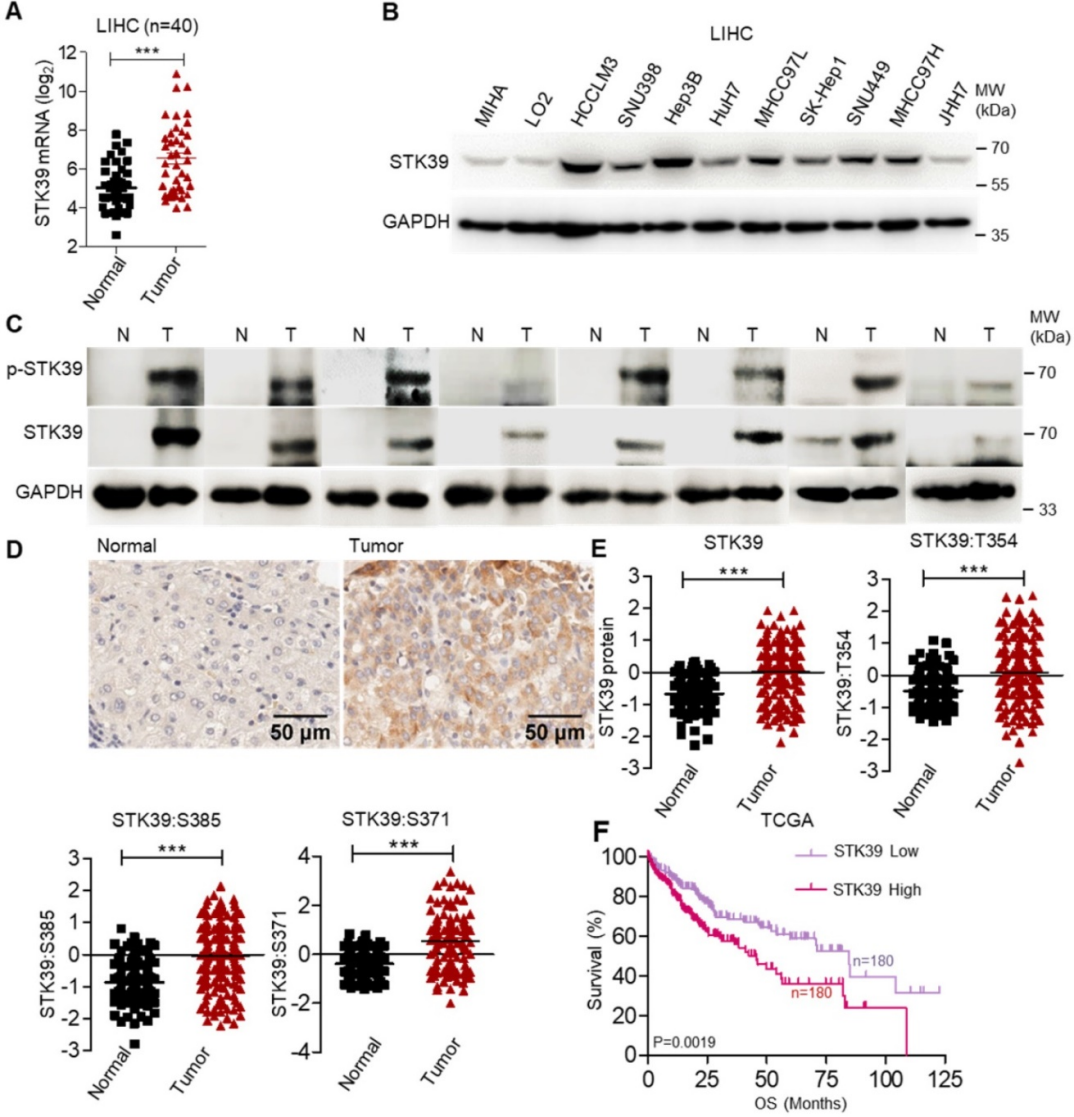
** STK39 up-regulation in HCC tissues is regulated by SP1. (A)** The analysis of STK39 gene expression in human HCC tissues and matched normal tissues. **(B)** STK39 protein expression in non-tumorigenic MIHA, LO2 and various HCC cell lines was assessed by immunoblotting. **(C)** The expression and phosphorylation of STK39 in human HCC tissues (T) and matched normal tissues (N) were assessed by immunoblotting.** (D)** Representative images of STK39 expression in HCC tissues by IHC staining, scale bar, 50 µm. **(E)** The elevated expression of STK39 and phosphorylation levels of STK39 were also shown in the integrated proteogenomic characterization of HBV related HCC. **(F)** The expression of STK39 was associated with the survival of HCC patients using OncoLnc-linked TCGA database**.** Data are shown as mean ±SEM. ***p<0.001.

To clarify the potential role of STK39 in HCC, the expression of STK39 between matched normal liver tissues and tumor tissues of HCC patients was analyzed, and we observed that STK39 expression was markedly upregulated in HCC tumor tissues (about 62.5% patients with overexpressed STK39) (Figure 1A). We also examined the expression of STK39 in The Cancer Genome Atlas (TCGA) dataset of HCC. As shown in Figure S1A, STK39 expression was increased in HCC tumor tissues compared with normal liver tissues. Additionally, the expression levels of STK39 in HCC cell lines were significantly higher than normal hepatocytes (MIHA and LO2) (Figure 1B and Figure S1B). To validate the data from databases and cell lines, we quantified STK39 mRNA levels, protein and phosphorylation levels in matched normal liver tissues and tumor tissues of HCC patients by qPCR and immunoblotting. The results showed that both the expression and the phosphorylation levels of STK39 were significantly increased in HCC tumor tissues (Figure 1C and Figure S1C). Consistent with these, Immunohistochemical (IHC) staining assay confirmed that STK39 expression was elevated in HCC tumor tissues compared to normal liver tissues (Figure 1D). Moreover, the elevated expression of STK39 and phosphorylation levels of STK39 were also shown in the integrated proteogenomic characterization of HBV-related HCC (Figure 1E) 36. However, pan-cancer analysis using TCGA dataset showed that overexpression of STK39 is mainly in liver cancer (both LIHC and CHOL) and few other cancer types (Figure S1D). Elevated expression of STK39 was negatively correlated with both overall survival (OS) and disease free survival (DFS) of HCC patients analyzed from the TCGA dataset (Figure 1F and Figure S1E). These observations showed that STK39 expression is significantly upregulated and associated with poor outcome of HCC.

To elucidate the underlying mechanisms of STK39 overexpressed in HCC, 2000 bp promoter regions of STK39 were analyzed with JASPAR and PROMO databases. We found two putative specificity protein 1 (SP1, overexpressed in human HCC as a previous study reported) binding sequences (CCGCCC) upstream to the TSS of the STK39 gene (Figure S1F) 37. To verify whether STK39 expression was regulated by SP1 in HCC, HCC cells (HuH7, HCCLM3 and Hep3B) were treated with SP1 inhibitor, Mithramycin A (MITA). We found that STK39 mRNA and protein expression levels were significantly reduced by MITA (Figure S1G-H). Similarly, SP1 knockdown by small interfering RNA (siRNA) in HCC cells significantly inhibited STK39 mRNA and protein expression (Figure S1I-J), whereas overexpression of SP1 in HCCLM3 cells dramatically promoted STK39 protein expression (Figure S1K). To further confirm that, the activity of the STK39 promoter was tested by luciferase reporter assay. As shown in Figure S1L, transcription of the luciferase gene controlled by the STK39 promoter was significantly activated by SP1. Taken together, our results demonstrate that STK39 is significantly upregulated in HCC and correlated with a poor outcome; in addition, the expression of STK39 in HCC was regulated by SP1.

### STK39 promotes proliferation and tumorigenesis of HCC cells

**Figure 2:**
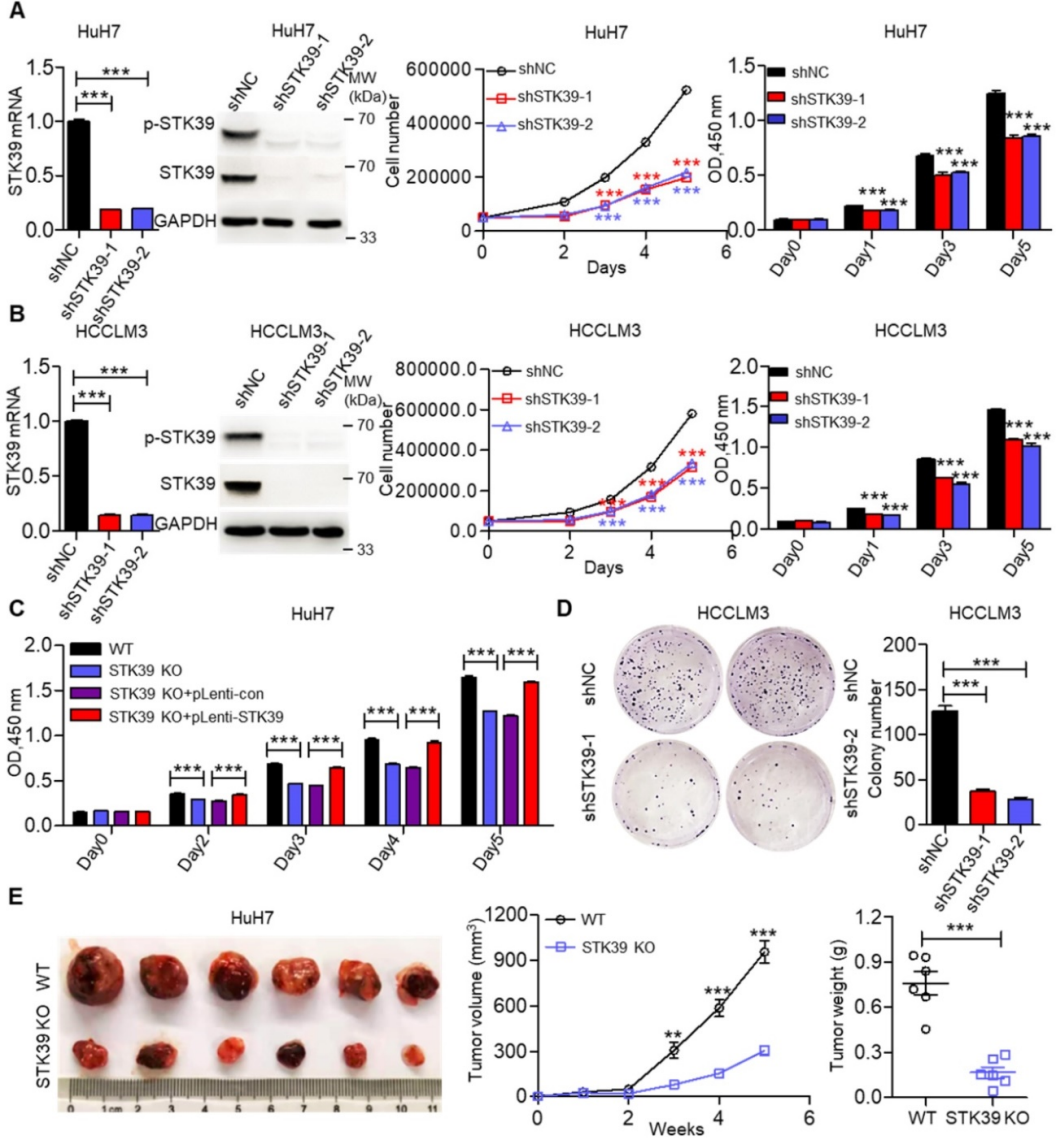
** STK39 overexpression promotes HCC proliferation. (A)** Stable knockdown of STK39 in HuH7 cells by shRNA, the expression and phosphorylation of STK39 were assessed by qPCR and immunoblotting, the growth or the viability of cells was measured by trypan blue staining or CCK8 assay.** (B)** Stable knockdown of STK39 in HCCLM3 cells by shRNA, the expression and phosphorylation of STK39 were measured by qPCR and immunoblotting, the growth or the viability of cells was measured by trypan blue staining or CCK8 assay.** (C)** Knockout of STK39 in HuH7 cells or re-expression of STK39 in STK39-knockout HuH7 cells, the viability of cells was measured by CCK8 assay.** (D)** Stable knockdown of STK39 in HCCLM3 cells by shRNA, the growth of cells was measured by colony formation assay. **(E)** STK39-knockout HuH7 cells were injected subcutaneously into BALB/c nude mice, the tumor volumes were measured every week, and the tumor weights were measured after five weeks of inoculation. Data are shown as mean ±SEM. **p<0.01; ***p<0.001.

To elucidate the biological functions of STK39 in HCC growth, we initially knocked down STK39 expression using siRNA in HuH7 cells, then the growth of cells was measured by trypan blue staining or CCK8 assay. As shown in Figure S2A, knockdown of STK39 significantly attenuated the growth of HuH7 cells. We then established stable STK39-knockdown HCC cell lines. We found that knockdown of STK39 in HCC cells by shRNA also dramatically decreased the proliferation of HCC cells (Figure 2A-B and Figure S2B). However, the growth of normal hepatocytes LO2 (STK39 low expression) was little affected after knockdown of STK39 (Figure S2C). To confirm this function of STK39, STK39-knockout HuH7 cell line was generated. We found that knockout of STK39 significantly suppressed cell growth while re-expressed STK39 in STK39-knockout HuH7 cells restored the growth of the cells (Figure 2C and Figure S2D). Consistent with these findings, the growth inhibitory effect of STK39 knockdown in HCC cells was demonstrated by colony formation assay (Figure 2D and Figure S2E). Moreover, in a 3D culture model, knockdown of STK39 impaired the growth of HCCLM3 cells while overexpression of STK39 enhanced the growth of HCCLM3 cells (Figure S2F). These results indicate that STK39 promotes the proliferation of HCC cells *in vitro*. To further determine whether STK39 had the same effect on HCC growth *in vivo*, STK39 knockdown or knockout HuH7 cells were injected subcutaneously at bilateral sites of nude mice. As shown in Figure 2E and Figure S2G, the volume and weight of tumors formed by STK39-knockout or STK39-knockdown HuH7 cells were dramatically slowed down in nude mice. All these results reveal that STK39 promotes the proliferation and tumorigenesis of HCC cells.

### STK39 deficiency enhances apoptosis and induces G2/M cell cycle arrest in HCC

**Figure 3:**
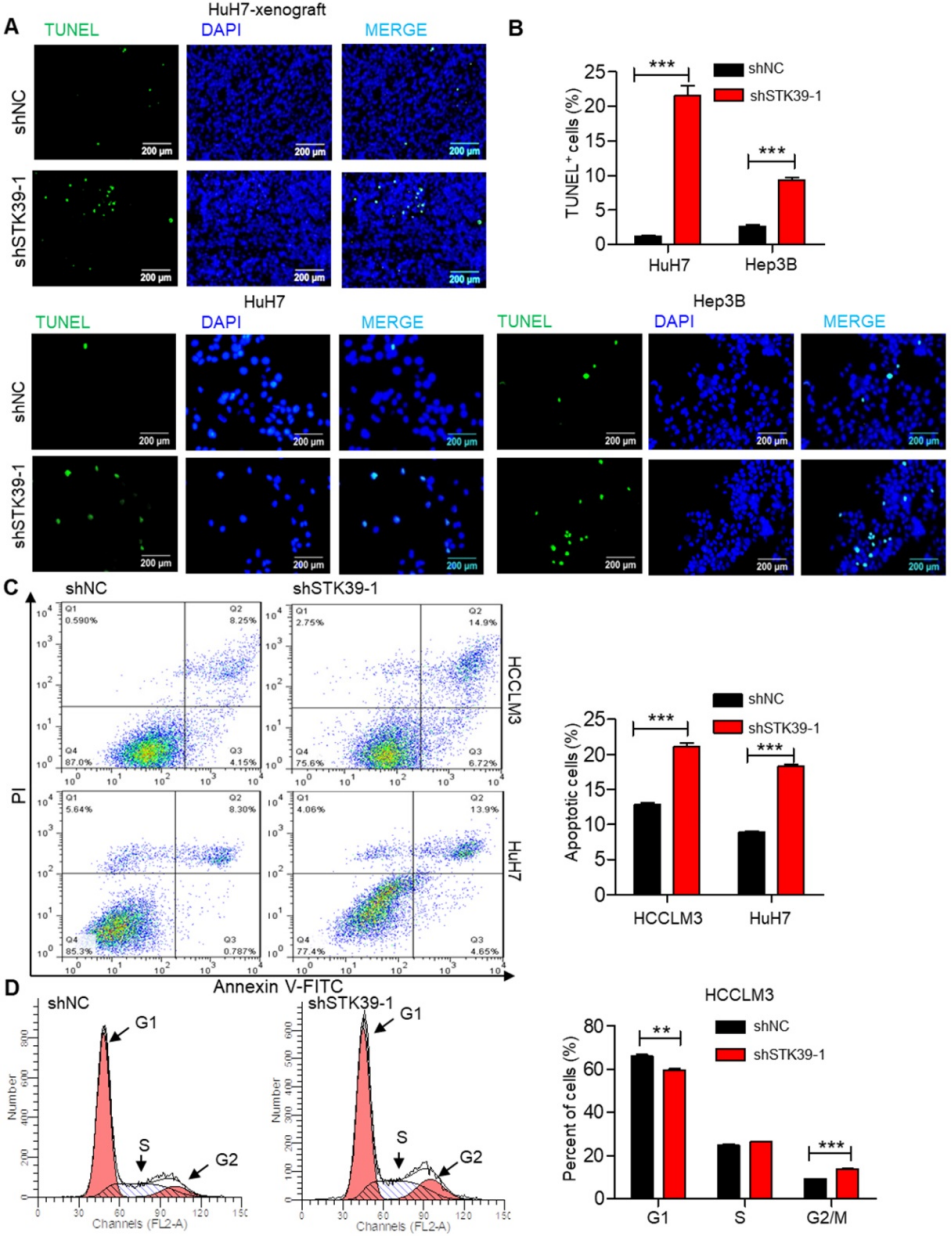
** Knockdown of STK39 induces HCC cells apoptosis and cell cycle arrest. (A)** TUNEL assay analysis of apoptosis in tumor xenografts generated by subcutaneous injection of STK39 stable knockdown cells in mice.** (B)** Stable knockdown of STK39 in HuH7 and Hep3B cells by shRNA, the percentage of apoptotic cells was examined by TUNEL assay.** (C)** Stable knockdown of STK39 in HCCLM3 and HuH7 cells by shRNA, the percentage of apoptotic cells was analyzed by Annexin V-FITC/PI staining assay.** (D)** Cell cycle analysis of STK39-knockdown and control HCCLM3 cells. Data are shown as mean ±SEM. **p<0.01; ***p<0.001.

Rapid cell division and apoptosis resistance are common features of the progression of tumors 38. Indeed, we set out to investigate the effect of STK39 on apoptosis and cell cycle of HCC. TUNEL assay analysis of STK39-knockdown HuH7 xenografts showed that knockdown of STK39 increased the level of apoptosis (Figure 3A). Moreover, the percentage of TUNEL-positive cells was also markedly increased after knockdown of STK39 in HuH7 and Hep3B cells (Figure 3B). To confirm that, STK39 knockdown-induced cancer cell apoptosis was further analyzed by Annexin V/propidium iodide analysis. As shown in Figure 3C, STK39 silencing dramatically increased the percentage of apoptosis in both HCCLM3 and HuH7 cells, clarifying that STK39 deficiency enhanced apoptosis in HCC cells. To understand the impact of STK39 on cell cycle, stable STK39-knockdown and control HCCLM3 cells were stained with PI and analyzed by flow cytometry. As shown in Figure 3D, knockdown of STK39 led to decreased cells at the G1 phase and increased cells at the G2/M phase. These findings collectively propose that STK39 deficiency enhances apoptosis and induces G2/M cell cycle arrest in HCC cells.

### STK39 promotes the migration, invasion and epithelial-mesenchymal transition (EMT) of HCC cells

**Figure 4:**
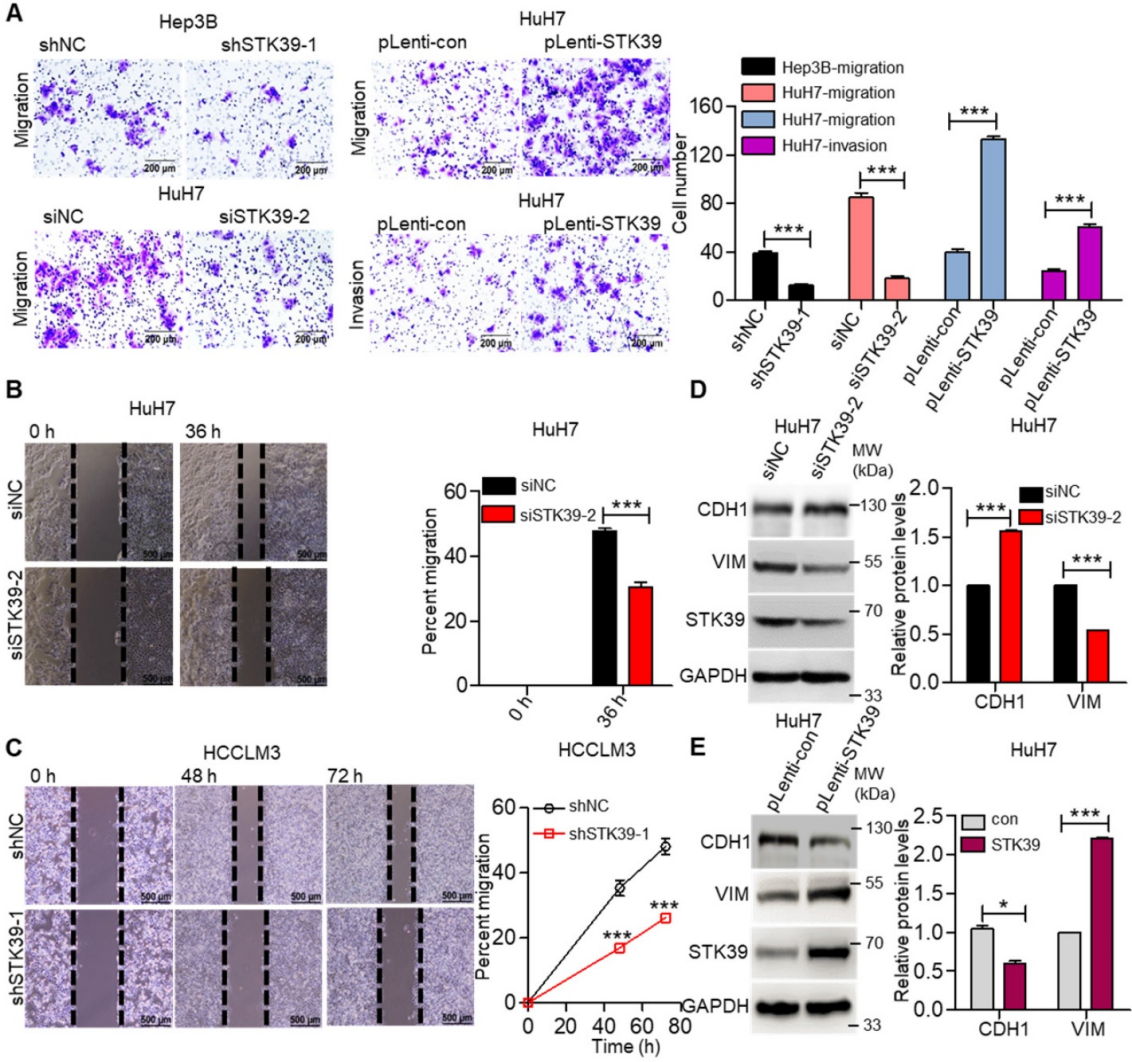
** STK39 promotes migration, invasion and EMT in HCC cells. (A)** Knockdown or overexpression of STK39 in HCC cells, invasive or migrated cells were measured by transwell assay with or without matrix.** (B)** Knockdown of STK39 in HuH7 cells by siRNA, cell migration ability was evaluated by wound healing assay.** (C)** Stable knockdown of STK39 in HCCLM3 cells by shRNA, cell migration ability was evaluated by wound healing assay.** (D)** Knockdown of STK39 in HuH7 cells by siRNA, expression of STK39, CDH1 and VIM were assessed by immunoblotting. **(E)** Overexpressing STK39 in HuH7 cells, expression of STK39, CDH1 and VIM were assessed by immunoblotting. Data are shown as mean ±SEM. *p<0.05; ***p<0.001.

Metastasis is one of the major obstacles to improve the prognosis of HCC. To explore the influence of STK39 on the metastasis of HCC, the migration, invasion, and wound healing abilities were examined. As shown in Figure 4A, knockdown of STK39 in Hep3B or HuH7 cells significantly suppressed the migration of the cells. Meanwhile, the forced expression of STK39 promoted the migration and invasion of HuH7 cells. In line with these findings, the wound healing assay also revealed that knockdown of STK39 attenuated the migration of HCC cells (Figure 4B-C). These data indicate that STK39 increased the motility of the HCC cells. EMT is a crucial step for the metastasis of cancer cells 39. Thus, we examined whether STK39 regulated the EMT of HCC cells. As shown in Figure 4D-E, STK39 knockdown by siRNA in HuH7 cells significantly increased the protein level of E-cadherin (CDH1) and decreased the protein level of vimentin (VIM). Conversely, overexpression of STK39 in HuH7 cells decreased the protein level of CDH1 and increased that of VIM. Collectively, our results demonstrate that STK39 endorses the migration, invasion and EMT of HCC cells.

### STK39 promotes HCC progression through activating ERK signaling pathway

**Figure 5:**
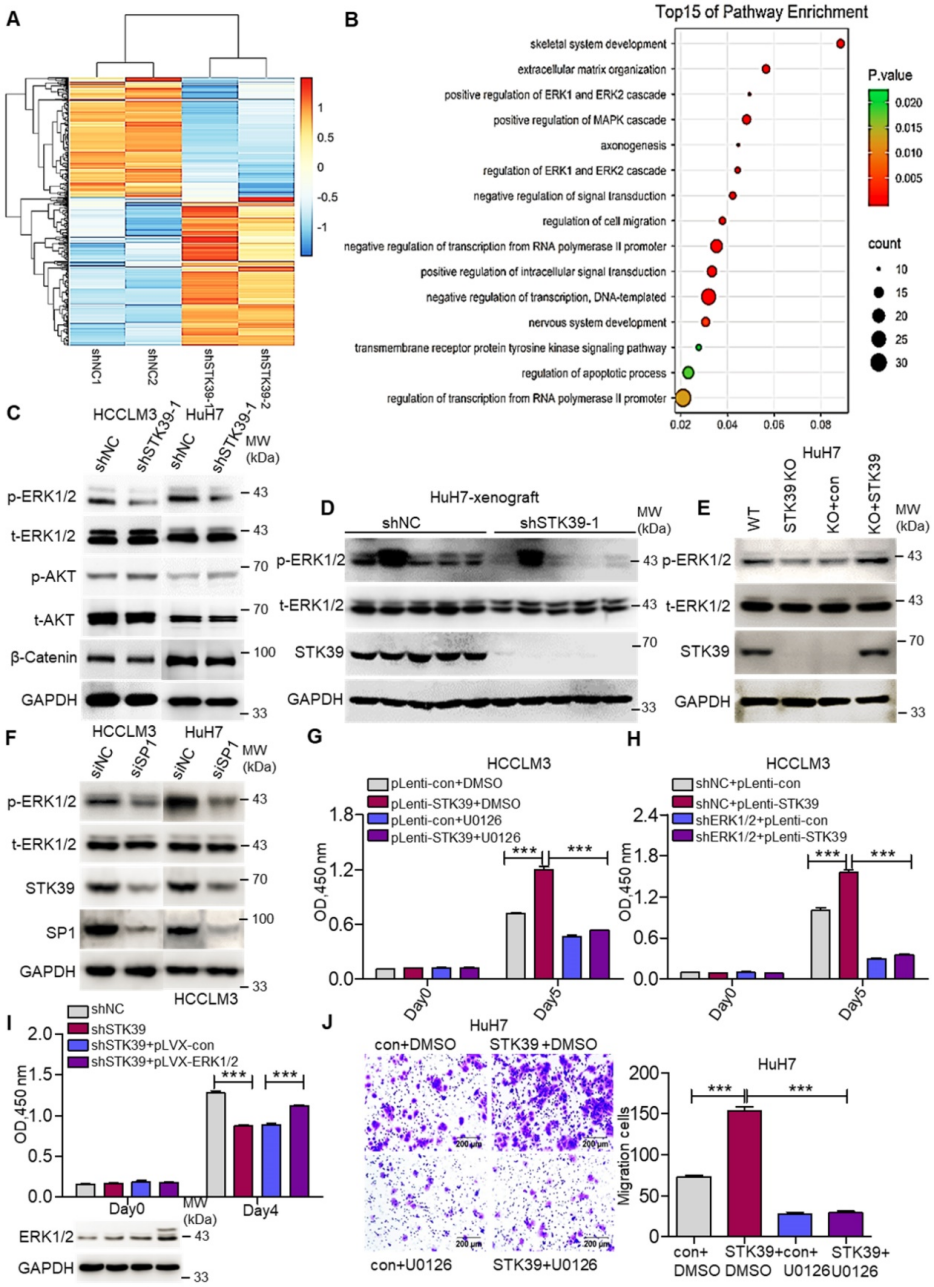
** STK39 mediates oncogenic effects on HCC cells through activating the ERK1/2 pathway. (A)** Stable knockdown of STK39 in HuH7 cells by shRNA, the differentially expressed genes were identified by RNA-sequence analysis.** (B)** Pathway enrichment analysis of differentially expressed genes in RNA-sequence data from (A).** (C)** Stable knockdown of STK39 in HCC cells by shRNA, the levels of p-ERK1/2, p-AKT and β-catenin were examined by immunoblotting.** (D)** Immunoblotting analysis of p-ERK1/2 level in tumor xenografts generated from subcutaneous inoculation of STK39 stable knockdown cells in mice.** (E)** STK39-/- HuH7 cells were reconstituted by infection of STK39 overexpression lentivirus, the levels of p-ERK1/2 and STK39 were assessed by immunoblotting. **(F)** HCC cells were transfected with negative control siRNA or SP1-specific siRNA for 72 h to knockdown SP1, the levels of p-ERK1/2, STK39 and SP1 were examined by immunoblotting. **(G)** STK39-overexpression and control HCCLM3 cells were treated with or without U0126, and the viability of cells was measured by CCK8 assay.** (H)** ERK1/2-knockdown and control HCCLM3 were infected with control or STK39 overexpression lentivirus, the viability of cells was measured by CCK8 assay.** (I)** Stable overexpression of ERK1/2 in STK39-knockdown HCCLM3 cells, the viability of cells was measured by CCK8 assay. **(J)** STK39-overexpression and control HuH7 cells were treated with or without U0126, migrated cells were measured by transwell assay. Data are shown as mean ±SEM. ***p<0.001.

We then inspected the molecular mechanism by which STK39 contributes to HCC progression. An RNA-sequence was performed using control HuH7 and STK39-knockdown HuH7 cells to analyze the differentially expressed genes. The genes whose expression down-regulated more than 2-fold were selected to analyze pathway enrichment. We found that the ERK1/2 signaling pathway had a high correlation with downstream of STK39 (Figure 5A-B and Figure S3A). Considering the pivotal role of MEK/ERK, PI3K/AKT and Wnt/β-Catenin signaling pathways for HCC progression, phosphorylated ERK1/2, phosphorylated AKT and levels of β-Catenin were further assessed by western blotting. As shown in Figure 5C and Figure S3B, knockdown of STK39 in HCC cells dramatically reduced the phosphorylation of ERK1/2, whereas the phosphorylation of AKT, levels of β-Catenin, total ERK1/2 and AKT were not significantly affected. Similarly, the phosphorylation of ERK1/2 in tumor xenografts, generated from subcutaneous inoculation of STK39-knockdown HuH7 cells, declined significantly (Figure 5D and Figure S3C-D). Further, we found that knockout of STK39 in HuH7 cells also significantly suppressed the phosphorylation of ERK1/2 while re-expressed STK39 restored this process (Figure 5E and Figure S3E). Moreover, when we knocked down SP1 to inhibit the expression of STK39 or knocked down WNK1 (which is an upstream activator of STK39) to suppress the phosphorylation of STK39 in HCC cells, the phosphorylation of ERK1/2 would be decreased subsequently (Figure 5F and Figure S3F-G) 40, 41. These results indicate that STK39 positively regulates the activation of the ERK signaling pathway. We then attempted to examine whether STK39 promotes HCC progression dependent on the ERK signaling pathway. As shown in Figure 5G and Figure S3H, overexpression of STK39 in HCCLM3 cells caused significant growth promotion and ERK1/2 phosphorylation. However, treating the cells with ERK1/2 inhibitor U0126 repressed STK39-mediated cell growth and ERK1/2 phosphorylation. Similarly, overexpression of STK39 in control HCCLM3 cells dramatically stimulated the growth of the cells and the phosphorylation of ERK1/2, while overexpression of STK39 in ERK1/2-knockdown HCCLM3 cells could not encourage the growth of the cells and the phosphorylation of ERK1/2 (Figure 5H and Figure S3I). Consistent with these, overexpression of ERK1/2 rescued the growth effect of STK39-knockdown cells (Figure 5I). In addition, we found that overexpression of STK39 highly provoked the migration of HuH7 cells, while ERK1/2 inhibitor U0126 eliminated this process (Figure 5J). Taken together, these data suggest that STK39 promotes HCC progression via activating ERK signaling pathway.

### STK39 interacts with PLK1 and regulates PLK1 phosphorylation

**Figure 6:**
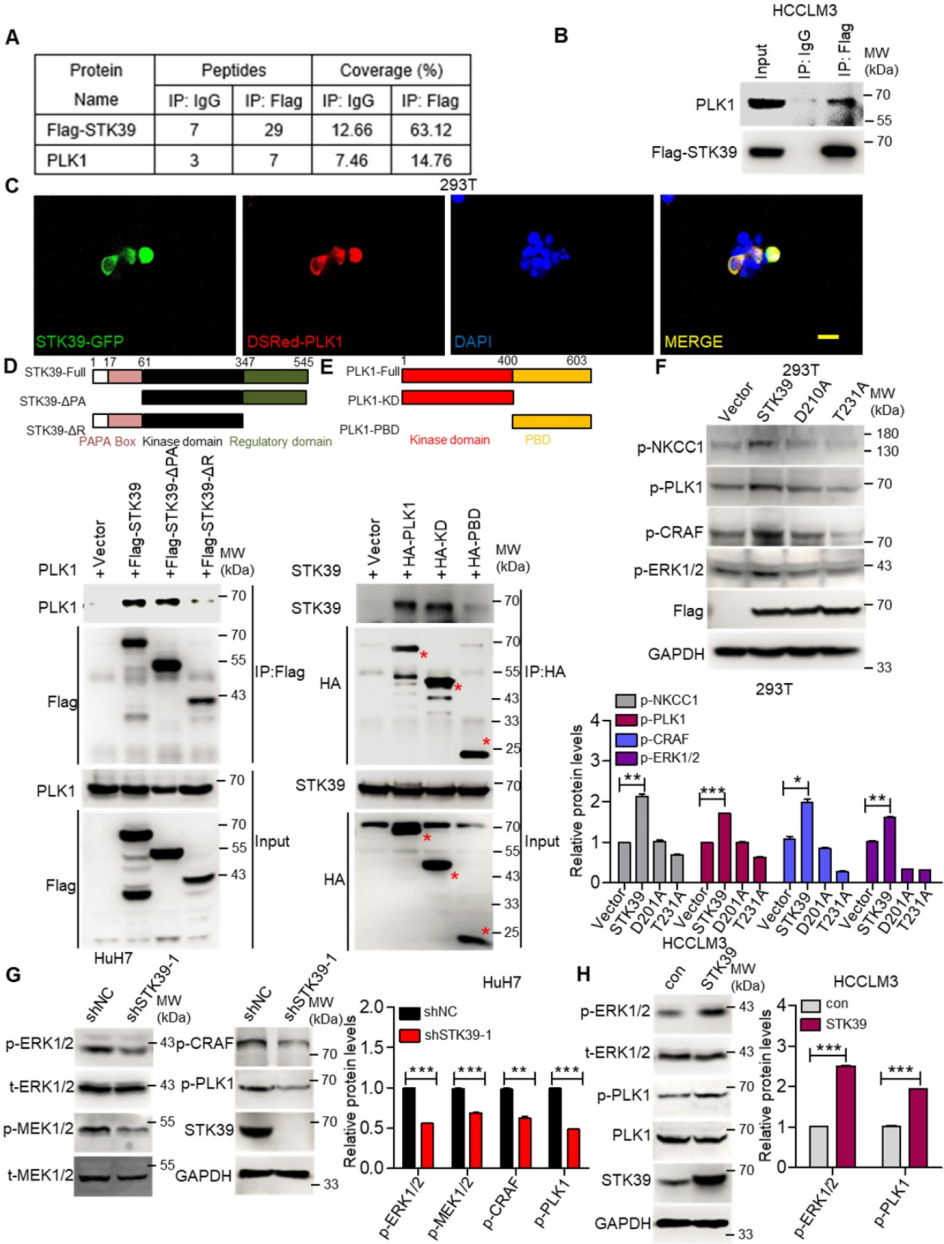
** STK39 interacts with PLK1 and promotes PLK1 phosphorylation. (A)** HCCLM3-Flag-STK39 cells were lysed and immunoprecipitated with anti-Flag antibody or control IgG, binding proteins were analyzed by mass spectrometry.** (B)** HCCLM3-Flag-STK39 cells were lysed and immunoprecipitated with anti-Flag antibody or control IgG, STK39-PLK1 interaction was assessed by immunoprecipitation and immunoblotting.** (C)** 293T cells were transfected with plasmids encoding STK39-GFP and Dsred-PLK1 for 72 h, co-localization of STK39 and PLK1 was analyzed by confocal microscopy, scale bar, 25 µm.** (D)** HA-PLK1 was co-transfected with Flag-STK39 or Flag-STK39-truncated mutants into 293T cells for 48 h, cell lysates were immunoprecipitated using anti-Flag antibody and immunoblotted with antibodies to PLK1 or Flag tags.** (E)** Flag-STK39 was co-transfected with HA-PLK1 or HA-PLK1-truncated mutants into 293T cells for 48 h, cell lysates were immunoprecipitated using anti-HA antibody and immunoblotted with antibodies to STK39 or HA tags. **(F)** 293T cells were transfected with plasmids encoding Flag-STK39, Flag-STK39 (D210A) and Flag-STK39 (T231A) for 48 h, the levels of p-NKCC1, p-PLK1, p-CRAF and p-ERK1/2 were assessed by immunoblotting. **(G)** Stable knockdown of STK39 in HuH7 cells by shRNA, the levels of p-ERK1/2, p-MEK1/2, p-CRAF, p-PLK1 and STK39 were examined by immunoblotting. **(H)** The levels of p-ERK1/2, p-PLK1 and STK39 in STK39-overexpression and control HCCLM3 cells were assessed by immunoblotting. Data are shown as mean ±SEM. *p<0.05; **p<0.01; ***p<0.001.

To further investigate how STK39 promotes the activation of the ERK signaling pathway, an immunoprecipitation (IP) assay was carried out to explore the potential STK39-binding proteins. After being analyzed by mass spectrometry (Supplementary Table S1-S2), we found that polo-like kinase 1 (PLK1) could bind to STK39 (Figure 6A and Figure S4A). As a serine/threonine kinase, PLK1 is overexpressed in a variety of human tumors and induces tumor progression. A previous study also showed that PLK1 motivates the activation of CRAF/ERK signaling via interacting with CRAF 42. To confirm the interaction between PLK1 and STK39, a co-immunoprecipitation assay was performed. As shown in Figure 6B, PLK1 specifically interacted with STK39. In addition, when plasmids encoding STK39-GFP and Dsred-PLK1 were transfected to 293T cells, co-localization of STK39 and PLK1 appeared by confocal microscopy (Figure 6C). To validate the specific region of STK39 that binds to PLK1, two mutants of STK39 were designed. The co-immunoprecipitation assay showed that the regulatory domain (347-545) of STK39 was responsible for its interaction with PLK1 (Figure 6D). Next, we attempted to define the specific binding region of PLK1 that interacts with STK39. As shown in Figure 6E, STK39 could interact with the kinase domain of PLK1 but not the polo-box domain (PBD), suggesting that the kinase domain of PLK1 is essential for the interaction with STK39. These results propose that PLK1 acts as a binding partner of STK39.

To determine whether the interaction between STK39 and PLK1 is responsible for the activation of the ERK signaling pathway, we first tested the influence of PLK1 on the activation status of the ERK signaling pathway in HCC. As shown in Figure S4B, knockdown of PLK1 by siRNA obviously suppressed the phosphorylation of CRAF and ERK1/2 in HuH7 cells. Similar results were observed in HCCLM3 cells after PLK1 knockdown by shRNA (Figure S4C). Additionally, treating HuH7 and HCCLM3 cells with a highly potent PLK1 inhibitor BI 6727 also strongly impaired the phosphorylation of CRAF, MEK1/2 and ERK1/2 (Figure S4D-E). Such results illustrate that PLK1 promotes the activation of the ERK signaling pathway in HCC. Next, we inspected whether STK39 regulates the activation of PLK1. As shown in Figure 6F, overexpression of STK39 in 293T cells obviously promotes the phosphorylation of NKCC1 (the phosphorylation of NKCC1 as a positive control), PLK1, CRAF and ERK1/2 43, 44. However, catalytically inactive STK39 (D210A) and WNK1 insensitive STK39 (T231A) could not facilitate the phosphorylation of PLK1 and ERK1/2 45, 46. Moreover, knockdown of STK39 in HuH7 cells significantly decreased PLK1, CRAF, MEK1/2 and ERK1/2 phosphorylation, while overexpression of STK39 in HCCLM3 cells enhanced these processes (Figure 6G-H). High STK39-expressed HCC cells also had an elevated PLK1 and ERK1/2 phosphorylation (Figure S4F). Taken together, these results indicate that STK39 interacts with PLK1 and regulates the phosphorylation of PLK1.

### STK39 mediates oncogenic effects on HCC cells via activating the PLK1-ERK1/2 pathway

**Figure 7:**
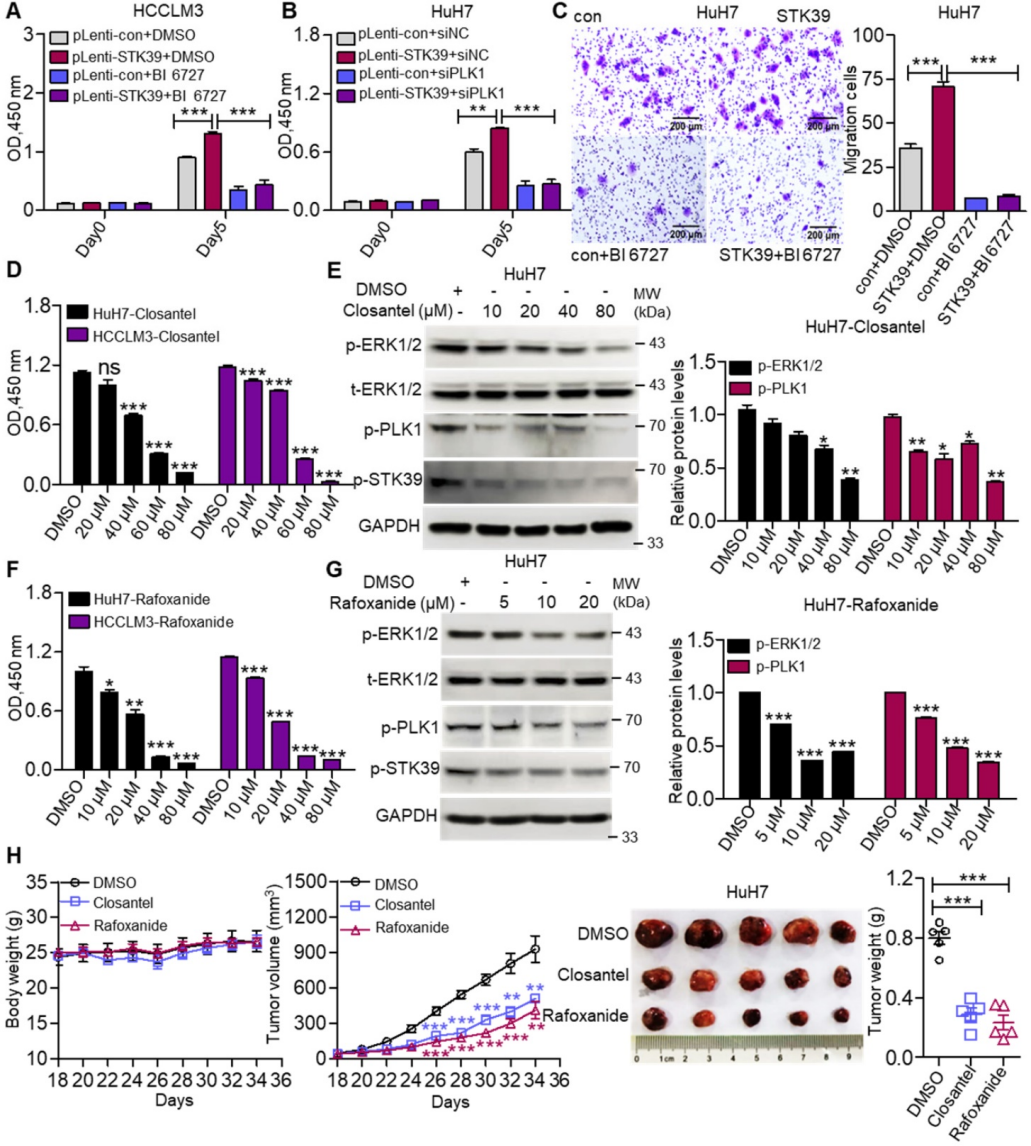
** STK39 promotes the progression of HCC via activating the PLK1-ERK1/2 pathway. (A)** STK39-overexpression and control HCCLM3 cells were treated with or without BI 6727, and the viability of cells was measured by CCK8 assay.** (B)** STK39-overexpression and control HuH7 cells were transfected with negative control siRNA or PLK1-specific siRNA for 48 h, the viability of cells was measured by CCK8 assay.** (C)** STK39-overexpression and control HuH7 cells were treated with or without BI 6727, migrated cells were measured by transwell assay. **(D)** HCC cells were treated with indicated concentrations of Closantel for four days, the viability of cells was measured by CCK8 assay. **(E)** HuH7 cells were treated with indicated concentrations of Closantel for 2 h, the levels of p-ERK1/2, p-PLK1 and p-STK39 were examined by immunoblotting. **(F)** HCC cells were treated with indicated concentrations of Rafoxanide for four days, the viability of cells was measured by CCK8 assay. **(G)** HuH7 cells were treated with indicated concentrations of Rafoxanide for 2 h; the levels of p-ERK1/2, p-PLK1 and p-STK39 were examined by immunoblotting. **(H)** HuH7 cells were injected subcutaneously into nude mice (n=5/group), after 18 days, mice were treated with or without 20 mg/kg STK39 inhibitors every two days for 16 days (Tumor sizes and the weights of mice were measured every two days). After 34 days, the mice were euthanized, and the tumor weights were measured. Data are shown as mean ±SEM. *p<0.05; **p<0.01; ***p<0.001; ns, not significant.

To investigate whether STK39 promotes the progression of HCC depending on PLK1, we first confirmed whether PLK1 aggravates the progression of HCC. As shown in Figure S5A-B, inhibition or knockdown of PLK1 significantly suppressed the growth of HCC cells. Moreover, PLK1 silencing intensely increased the percentage of apoptosis in both HuH7 and HCCLM3 cells and led to cell cycle arrested at the G2/M phase (Figure S5C-E). These results suggest that PLK1 encourages the progression of HCC. To further verify whether STK39 mediated oncogenic effects on HCC dependent on PLK1, STK39-overexpression and control HCCLM3 cells were treated with or without BI 6727. As shown in Figure 7A, overexpression of STK39 in HCCLM3 cells significantly raised the growth of the cells. However, treating the cells with PLK1 inhibitor BI 6727 reduced the STK39-mediated cell growth. Consistently, the overexpression of STK39 in control HuH7 cells drastically accelerated cell growth while PLK1 knockdown in STK39-overexpressed HuH7 cells decelerated this process (Figure 7B). Moreover, we noticed that overexpression of STK39 remarkably promoted the migration of HuH7 cells, while PLK1 inhibitor BI 6727 demoted this process (Figure 7C). Thus, these results demonstrate that STK39 positively regulates the progression of HCC depending on PLK1.

To evaluate the importance of STK39/PLK1/ERK1/2 axis in the progression of HCC, two STK39 inhibitors (Closantel and Rafoxanide) were selected to treat HCC cells. Data showed that STK39 inhibitors significantly decreased the cell viability of HCC cells and inhibited the phosphorylation of STK39, PLK1 and ERK1/2 (Figure 7D-G) 43, 47. To further evaluate the therapeutic potential of STK39 inhibitors* in vivo*, the mouse xenograft model was used. As shown in Figure 7H, STK39 inhibitors obviously reduced the growth of HCC cells-induced tumors in BALB/c nude mice, but had little influence on the mice bodyweight. In summary, our results demonstrate that STK39 promotes the progression of HCC is dependent on PLK1-mediated activation of the ERK signaling pathway, and the STK39/PLK1/ERK1/2 axis may be a potential drug target for HCC.

## Discussion

HCC is one of the most lethal malignancies worldwide and causes an estimated 800,000 deaths annually 48. Although great efforts are made in the diagnosis and treatment of HCC, the treatment effect of HCC remains discouraging. Investigating the underlying molecular mechanisms of HCC and developing new therapeutic strategies is urgent. As a serine/threonine kinase, STK39 was previously demonstrated to interact with cation chloride cotransporters and regulate ion homeostasis, which is crucial for modulating renal salt transport and blood pressure 32, 49. Some studies also revealed that STK39 has an important role in regulating inflammatory diseases 50, 51. In recent years, the role of STK39 in tumorigenesis has been increasingly emphasized 35. However, the biological behavior and regulatory mechanism of STK39 in HCC remains unknown. In this study, we first discovered that STK39 was significantly upregulated in HCC and associated with a poor outcome. By analyzing of the promoter regions of STK39, we found that transcription factor SP1 contributed to STK39 dysregulation in HCC. We further demonstrated that overexpression of STK39 promoted the growth, metastasis and EMT of HCC, while knockdown of STK39 caused G2/M cell cycle arrest and induced apoptosis in HCC. Therefore, our results indicated that STK39 has an oncogenic role in HCC and may be a potential target for HCC treatment.

Emerging studies have proved that STK39 participates in tumorigenesis 33, 34, 52. However, its precise mechanism remains largely elusive. To explore the downstream signaling of STK39 in HCC, an RNA-sequence was performed, and the genes whose expression down-regulated more than 2-fold were selected to analyze pathway enrichment. According to pathway analysis, we found that ERK signaling was downstream of STK39. We, therefore, speculated that STK39 might activate ERK signaling pathway in HCC. Our western blotting results validated this speculation. MAPK/ERK signaling pathway plays a central role in the occurrence and development of HCC. Activated MAPK/ERK signaling enhances growth, metastasis and metabolism of HCC 53, 54. Blocking or knockdown of ERK1/2 eliminates the functions of STK39 in HCC, suggesting that STK39 endorses HCC progression via activating ERK signaling pathway.

As a key regulator of cell division and DNA damage response, PLK1 was reported to be implicated in the development of various cancers, including HCC 3, 55-58. However, the relationship between STK39, PLK1 and ERK signaling pathway remains to be clarified. In our study, to investigate how STK39 promotes the activation of the ERK signaling pathway, the protein complex of STK39 was analyzed by mass spectrometry and it was found that PLK1 could bind to STK39; the regulatory domain (347-545) of STK39 and the kinase domain of PLK1 contributed to this interaction. A previous study revealed that PLK1 promotes the activation of CRAF/ERK signaling via interacting with CRAF, and then leading to the EMT process 42. Thus, we studied the functional significance of the interaction between STK39 and PLK1 and found that STK39 is an upstream regulator of PLK1 and promotes its phosphorylation. Inhibition or knockdown of PLK1 eliminates the functions of STK39 in HCC, suggesting that STK39-promoted HCC progression is dependent on PLK1.

## Conclusions

In summary, our results reveal a novel role of STK39 in HCC. Elevated STK39 level in HCC patients is induced by transcription factor SP1 and correlates with poor clinical outcomes. STK39 promotes the growth and metastasis of HCC cells via interaction with PLK1 and activates the PLK1/ERK signaling axis. STK39/PLK1/ERK signaling axis may be a potential therapeutic target for HCC.

## Significance Statement

HCC has a poor prognosis, and the detailed molecular mechanism underlying HCC largely remains to be elucidated. In the current study, we found that STK39 is a novel oncogenic kinase, which is significantly elevated and activated in human HCC and contributes to HCC tumorigenesis through activation of the PLK1/ERK signaling pathway. These findings indicate that STK39 has an oncogenic role in HCC, and it may be a potential target for HCC treatment.

## Supplementary Material

Supplementary figures and tables.

## Abbreviations

HCC: hepatocellular carcinoma
IHC: immunohistochemistry
RT-PCR: reverse transcription PCR
shRNA: short hairpin RNA
STK39: Serine/threonine kinase 39
HBV: hepatitis B virus
HCV: hepatitis C virus
MAPK: Mitogen-activated protein kinase
ERK: extracellular signal-regulated kinase
PI3K: phosphoinositide 3-kinase
AKT: protein kinase B
IGFR: insulin-like growth factor receptor
EGFR: epidermal growth factor receptor
PLK1: polo-like kinase 1
PBD: polo-box domain
TCGA: The Cancer Genome Atlas
SP1: specificity protein 1
siRNA: small interfering RNA
TUNEL: terminal deoxynucleotidyl transferase dUTP nick end labeling
PI: propidium Iodide
EMT: epithelial-mesenchymal transition
IP: immunoprecipitation
LIHC: liver hepatocellular carcinoma
CHOL: cholangiocarcinoma
