# Supplementary material for: STK39 is a novel kinase contributing to the progression of hepatocellular carcinoma by the PLK1/ERK signaling pathway
Source: Theranostics. 2021 Jan 1;11(5):2108–22. doi: 10.7150/thno.48112 (PMC7797677; doi:10.7150/thno.48112)
Supplement: Supplementary file 1 — Supplementary figures and tables. [file thnov11p2108s1.pdf]

STK39 is a novel kinase contributing to the progression of hepatocellular carcinoma by the PLK1/ERK signaling pathway

Supplementary materials and methods

Reagents

CCK8，trypan blue and TUNEL kit were purchased from YEASEN, and crystal violet was purchased from Sangon Biotech. Dual-luciferase assay reagent was purchased from Promega. U0126, BI 6727, Closantel and Rafoxanide were purchased from MedChemExpress (MCE). Anti-STK39 antibodies were obtained from Abcam and sigma. Phospho-STK39 (Ser371) and phospho-NKCC1(Thr212/Thr217) antibodies were obtained from sigma. SP1, phospho-MEK1/2 (Ser217/Ser221), MEK1/2 antibodies were obtained from ABclonal. Anti-β-Catenin antibody was obtained from Santa Cruz Biotechnology. ERK1/2, phospho-p44/42 MAPK (ERK1/2) (Thr202/Tyr204), AKT, and phospho-AKT (Ser 473), phospho-PLK1 (Thr210) antibodies were obtained from Cell Signaling Technology. Phospho-CRAF (Ser 338) antibody was purchased from Sangon Biotech. An anti-vimentin antibody, anti-E-cadherin antibody, WNK1 antibody, anti-PLK1 antibody, anti-Flag antibody, anti-HA antibody, and anti-GAPDH antibody were purchased from Proteintech. Goat Anti-Rabbit IgG (H+L) HRP and Goat Anti-Mouse IgG (H+L) HRP secondary antibodies were purchased from Bioworld.

Clinical Samples

Eighteen freshly frozen HCC tissues and adjacent normal tissues were obtained from Henan Provincial People's Hospital and then subjected to STK39 mRNA detection. Eight HCC tissues and adjacent normal tissues were used for western blotting analysis. Paraffin sections from 50 HCC tissues were obtained from the Affiliated Drum Tower Hospital of Nanjing University Medical School and subjected to immunohistochemical staining.

Cell Lines and Culturing

The human HCC cell lines HuH7, Hep3B, HCCLM3, SNU398, MHCC97L, MHCC97H, SNU449, JHH7, SK-Hep1 and LO2, MIHA, 293T cells were obtained from the Cellbank of China (https://www.cellbank.org.cn/), American Type Culture Collection (ATCC), Korean Cell Line Bank (KCLB), Japanese Collection of Research Bioresources (JCRB). Cells were cultured in DMEM containing 10% FBS, 1% penicillin/streptomycin.

Plasmids, lentivirus preparation and stable cell lines

pLKO.1-shScramble, pLKO.1-shSTK39, pLKO.1-ERK1/2 and pLKO.1-PLK1 (validated) vectors were purchased from Sigma-Aldrich (St. Louis, MO, USA). pLenti-CRISPR V2, pMDLg/pRRE, pRSV-Rev and pMD2.G plasmids were obtained from Addgene. pLenti-CMV-GFP/puro, pLenti-CMV-STK39 and pLenti-CMV-SP1 vectors were purchased from Biogot Technology. pCDNA3.1-HA-PLK1,

pLVX-ERK1, pLVX-ERK2 were obtained from Miaoling Biotechnology. Flag-STK39, Flag-STK39-ΔPA, and Flag-STK39-ΔR truncate were cloned into pLenti-CMV-GFP/puro, HA-PLK1-KD and HA-PLK1-PBD truncate were cloned into pCDNA3.1. STK39-GFP and DSRed-PLK1 were cloned into pCDNA3.1. All vectors were sequenced.

To produce lentivirus, 293T cells were seeded into 6-well plates (6×10^5 cells per well) overnight. 2 μg of the lentiviral vector (pLKO.1-vector, pLenti-CMV-vector or pLenti-CRISPR V2 vector), 1 μg of pMDLg/pRRE, 0.4 μg of pRSV-Rev and 0.6 μg of pMD2.G were co-transfected into 293T cells using PEI. After 48 h, cell supernatants were collected and filtered through a 0.45-μm membrane (Millipore).

To generate stable cell lines, cells were seeded into 12-well plates (1×10^5 cells per well) overnight and infected with lentivirus for 48 h. The cells were then treated with 2 μg/mL of puromycin about 1 week before being used in experiments.

siRNAs and transfections

HuH7, HCCLM3 and Hep3B cells were seeded into 6-well plates (2×10^5 cells per well) overnight and then transfected with 60 nM siRNA (GenePharma) using RNAi-Mate (GenePharma) according to the manufacturer's instructions. The sequences of siRNA were: negative control, sense, 5′-UUCUCCGAACGUGUCACGUTT-3′, antisense, 5′-ACGUGACACGUUCGGAGAATT-3′; SP1, sense, 5′-GGUAGCUCUAAGUUUUG AUTT-3′, antisense,

5′-AUCAAAACUUAGAGCUACCTT-3′; STK39-1, sense, 5′-CCCACCCAAUGCUAAUGAATT-3′, antisense, 5′-UUCAUUAGCAUUGGGUGG GTT-3′; STK39-2, sense, 5′-GAGGUUCAAUGUUGGAUAUTT-3′, antisense, 5′-AUAUCCAACAUUGAACCUCTT-3′; PLK1, sense, 5′-AGAUCACCCUCCUUAAA UATT-3′, antisense, 5′-UAUUUAAGGAGGGUGAUCUTT-3′; WNK1-1, sense, 5′-CAGACAGUGCAGUAUUCACTT-3′, antisense, 5′-GUGAAUACUGCACUGUCU GTT-3′; WNK1-2, sense, 5′-GGAUCAAGUGCGAGAAAUUTT-3′, antisense, 5′-AA UUUCUCGCACUUGAUCCTT-3′.

Quantitative real-time PCR

Human HCC samples or cells were lysed in TRIzol reagent (TianGen), and cDNA was synthesized from extracted total RNA using 5×All-In-One RT MasterMix (ABMgood) according to the manufacturer’s protocol. Quantitative real-time PCR was performed with SYBR Green Master Mix (YEASEN), and 250 ng cDNA was used as a template. GAPDH was used as an internal control gene. The sequence-specific primers are as following: STK39, forward 5′-TTCATAAAACCGAAGACGGG-3′ and reverse 5′-GTATTTGTTCGGGGATGGTG-3′; SP1, forward 5′-CACTCTCACACCCATTGCCT -3′ and reverse 5′-CTTCCTCTCCACCTGCTGTG-3′; GAPDH, forward 5′-ACCCAG

AAGACTGTGGATGG-3′ and reverse 5′-TTCAGCTCAGGGATGACCTT-3′.

IHC

Paraffin-embedded tissue was cut into 6 μm slices. Subjected to antigen retrieval and endogenous peroxidases were blocked. Sections were incubated with the primary antibody overnight at 4 °C and then incubated with corresponding secondary antibody for 2 h at room temperature. Then, the sections were visualized with DAB, and nuclei were counterstained with hematoxylin. The tissue sections were visualized under a bright-field microscope (Leica Microsystems).

Cell proliferation assay

For CCK8 assay, STK39-knockdown or knockout cells were seeded in 96-well plates (2×10^3 cells per well), after 1, 3 and 5 days, 10 μL CCK8 was added to each well and incubated at 37 °C for 1.5 h. After incubation, the absorbance value was measured at 450 nm.

For cell counting assay, STK39-knockdown cells were seeded in 12-well plates (5×10^4 cells per well). On the indicated day, cells were collected and counted using a cell counter (JIMBIO) after staining with trypan blue.

For colony formation assay, STK39-knockdown cells were seeded in 6-well plates (2×10^3 cells per well), and the medium was refreshed every 3 days. After 15 days, the medium was removed, and the cells were fixed with 4% paraformaldehyde in PBS for 30 min at room temperature. Then the cells were stained with crystal violet for 30 min

and washed with PBS before being photographed.

For the 3D culture model, 24-well plates were covered with 200 μL of Matrigel (Corning) and maintained at 37 °C for 1 h for gel solidification. Then 2,000 HCCLM3 cells were seeded into each well and cultured in 800 uL completed DMEM for 10 days. 3D-cancer spheroids were observed under an inverted microscope, and relative volumes of 3D-cancer spheroids were measured using Image J software.

Cell migration and invasion assay

Cell migration and invasion assay were performed using a transwell system with or without Matrigel (Corning). Briefly, cells were starved for 12 h, and 5×10^4 cells suspended in DMEM without FBS plated on the upper chamber membranes. The chambers were then transferred to a well containing 600 μL medium with 20% FBS. After 36 h incubation, noninvasive cells were removed by cotton swabs. Invasive cells were fixed with 4% paraformaldehyde in PBS for 30 min at room temperature and stained with 0.1% crystal violet for 30 min. Five fields were imaged under a bright-field microscope, and the number of the cell was counted.

Wound-healing assay

STK39-knockdown cells were seeded in 6-well plates at a suitable density with three replicates and wounded by manual scratching with a 200-μL pipette tip. The cells were then washed with PBS and incubated at 37 °C in DMEM containing 1% FBS, 1% penicillin/streptomycin. The wound was photographed under an inverted microscope

at the indicated time points.

Apoptosis

Cellular apoptosis was measured using TUNEL (YEASEN) and Annexin V-FITC/PI Apoptosis Detection Kit (YEASEN). For TUNEL assay, cells (fixed with 4% paraformaldehyde in PBS for 30 min at room temperature) and paraffin-embedded tissue were processed for TUNEL staining according to the manufacturer's instructions.

For Annexin V-FITC/PI staining assay, cells were stained with fluorescein isothiocyanate (FITC) Annexin V and propidium iodide (PI) and then detected by flow cytometry (BD FACSCalibur). Data were analyzed with FlowJo software (Treestar).

Cell cycle analysis

STK39-knockdown cells were seeded in 6-well plates (1×10^5 cells per well) with three replicates. After 48 h, cells were collected and washed twice with PBS and fixed with ice-cold 75% ethanol overnight. The cells were washed twice with PBS and treated with RNase A for 30 min at 37°C. Following this, the cells were then stained with PI and analyzed by flow cytometry. The percentage of cells in different cell cycle phases was analyzed with ModFit software.

Luciferase Reporter Assay

293T cells were plated in 12-well plates (3×10^5 cells per well) overnight and co-transfected with reporter plasmid, renilla plasmid and SP1 overexpression plasmid for 30 h. The cells were lysed, and the luciferase activity was measured with a dual-luciferase reporter assay system (Promega) according to the manufacturer's instructions.

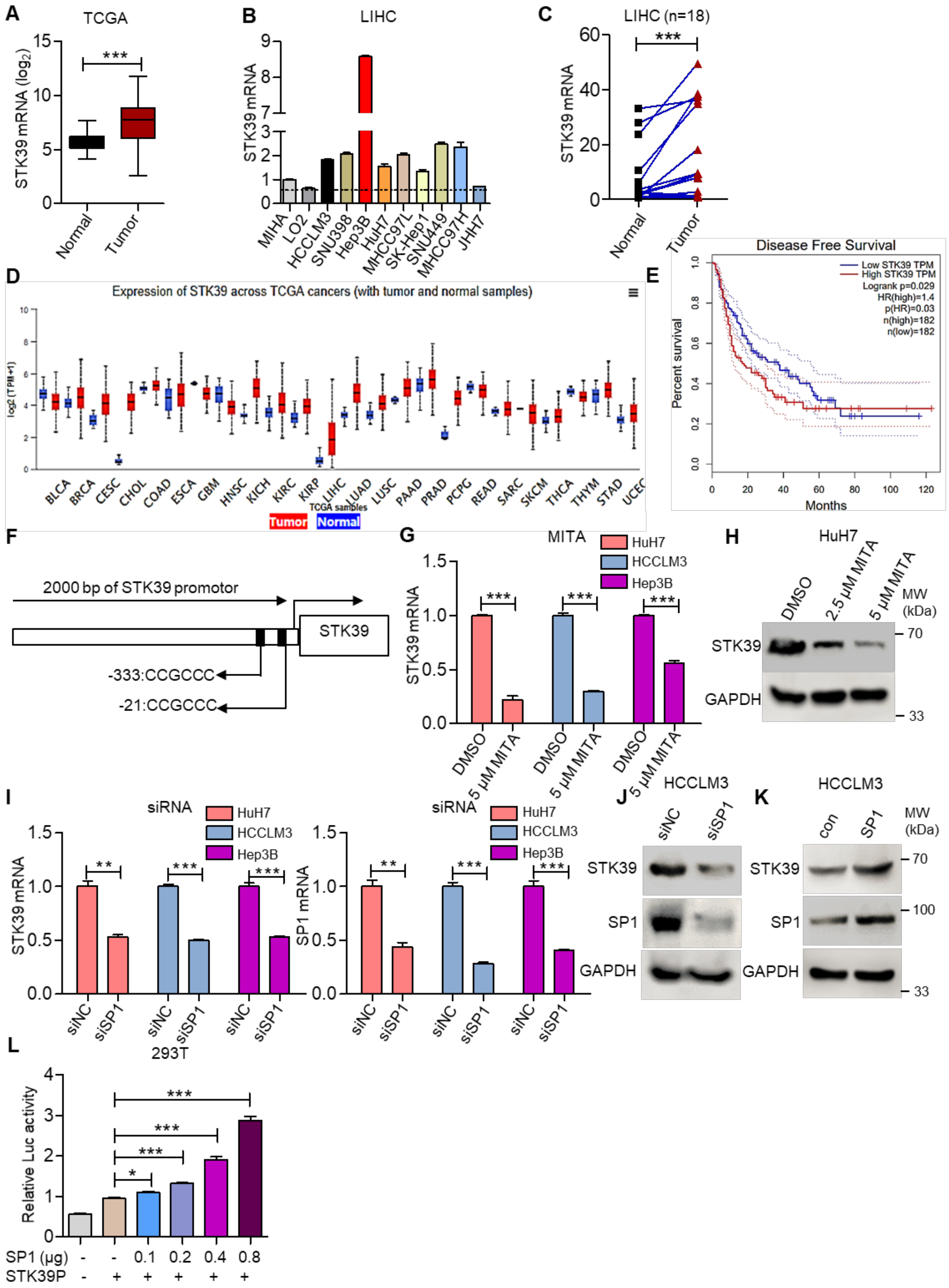

Figure S1. STK39 is upregulated in HCC, related to Figure 1. (A) Analysis of STK39 gene expression in human HCC tissues (T) and normal liver tissues (N) in TCGA database; (B) STK39 mRNA expression in non-tumorigenic MIHA, LO2 and various HCC cell lines was detected with qPCR; (C) STK39 mRNA expression in

human HCC tissues (T) and matched normal tissues (N) was detected with qPCR; (D) pan-cancer analysis using TCGA dataset showed that overexpression of STK39 is mainly in liver cancer (both LIHC and CHOL) and few other cancer types; (E) Elevated expression of STK39 was negatively correlated with disease free survival (DFS) of HCC patients analyzed from the TCGA dataset; (F) Two SP1 putative binding sites in the promoter region of STK39 were identified by analyzed with JASPAR and PROMO databases; (G) HCC cells were treated with or without SP1 inhibitor Mithramycin A (MITA, 5 μM) for 12 h, STK39 mRNA expression was analyzed by qPCR; (H) HuH7 cells were treated with or without SP1 inhibitor MITA (2.5 and 5 μM) for 24 h and STK39 protein expression was assessed by immunoblotting; (I) HCC cells were transfected with negative control siRNA or SP1-specific siRNA for 72 h, STK39 and SP1 mRNA expressions were analyzed by qPCR; (J) HCCLM3 cells were transfected with negative control siRNA or SP1-specific siRNA for 72 h, STK39 and SP1 protein expressions were assessed by immunoblotting; (K) STK39 protein expression in HCCLM3-con and HCCLM3-SP1 cells were assessed by immunoblotting; (L) 293T cells were co-transfected with PGL3 vector or pGL3-STK39 reporter plasmid, Renilla plasmid and SP1 overexpression plasmid for 30 h, luciferase activities were measured with a dual-luciferase reporter assay system. Data are shown as mean ±SEM. *$p<0.05$; **$p<0.01$; ***$p<0.001$.

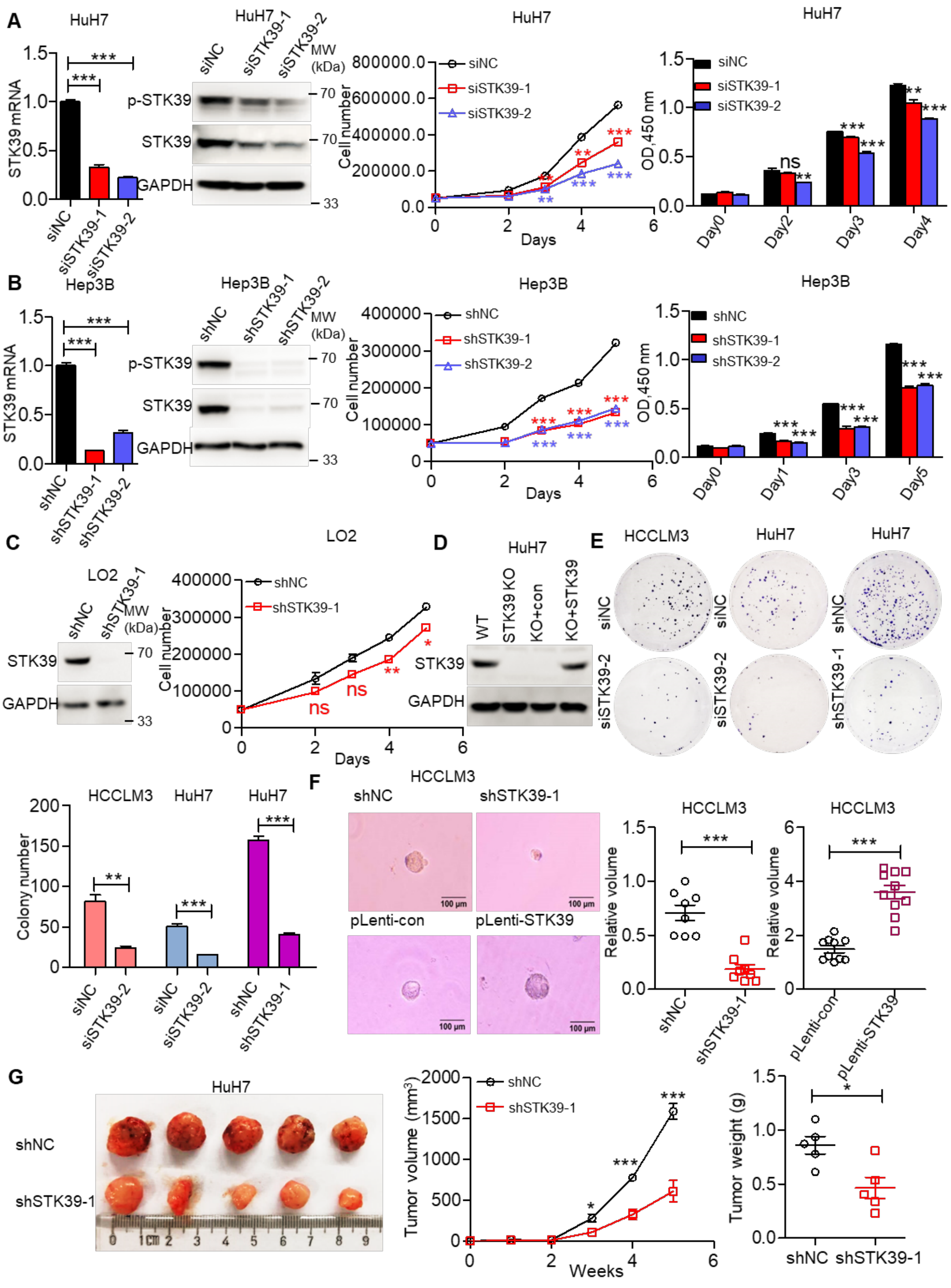

Figure S2. STK39 promotes the growth of HCC cells, related to Figure 2. (A) HuH7 cells were transfected with negative control siRNA or STK39-specific siRNA for 48 h to knockdown STK39, the expression and phosphorylation of STK39 were assessed by qPCR and immunoblotting, the growth or the viability of cells was

measured by trypan blue staining or CCK8 assay; (B) Stable knockdown of STK39 in Hep3B cells by shRNA, the expression and phosphorylation of STK39 were assessed by qPCR and immunoblotting, the growth or the viability of cells was measured by trypan blue staining or CCK8 assay; (C) Stable knockdown of STK39 in LO2 cells by shRNA, the growth of cells was measured by trypan blue staining assay; (D) STK39-/- HuH7 cells were reconstitution by infection of STK39 lentiviral and STK39 protein expression was assessed by immunoblotting; (E) Knockdown of STK39 in HCC cells, the growth of cells was measured by colony formation assay; (F) Stable knockdown or overexpression of STK39 in HCCLM3 cells, the growth of cells was evaluated by 3D culture; (G) Knockdown of STK39 in HuH7 cells and injected subcutaneously into BALB/c nude mice, the tumor volumes were measured every week, and the tumor weights were measured after five weeks inoculation. Data are shown as mean ±SEM. *$p<0.05$; **$p<0.01$; ***$p<0.001$; ns, not significant.

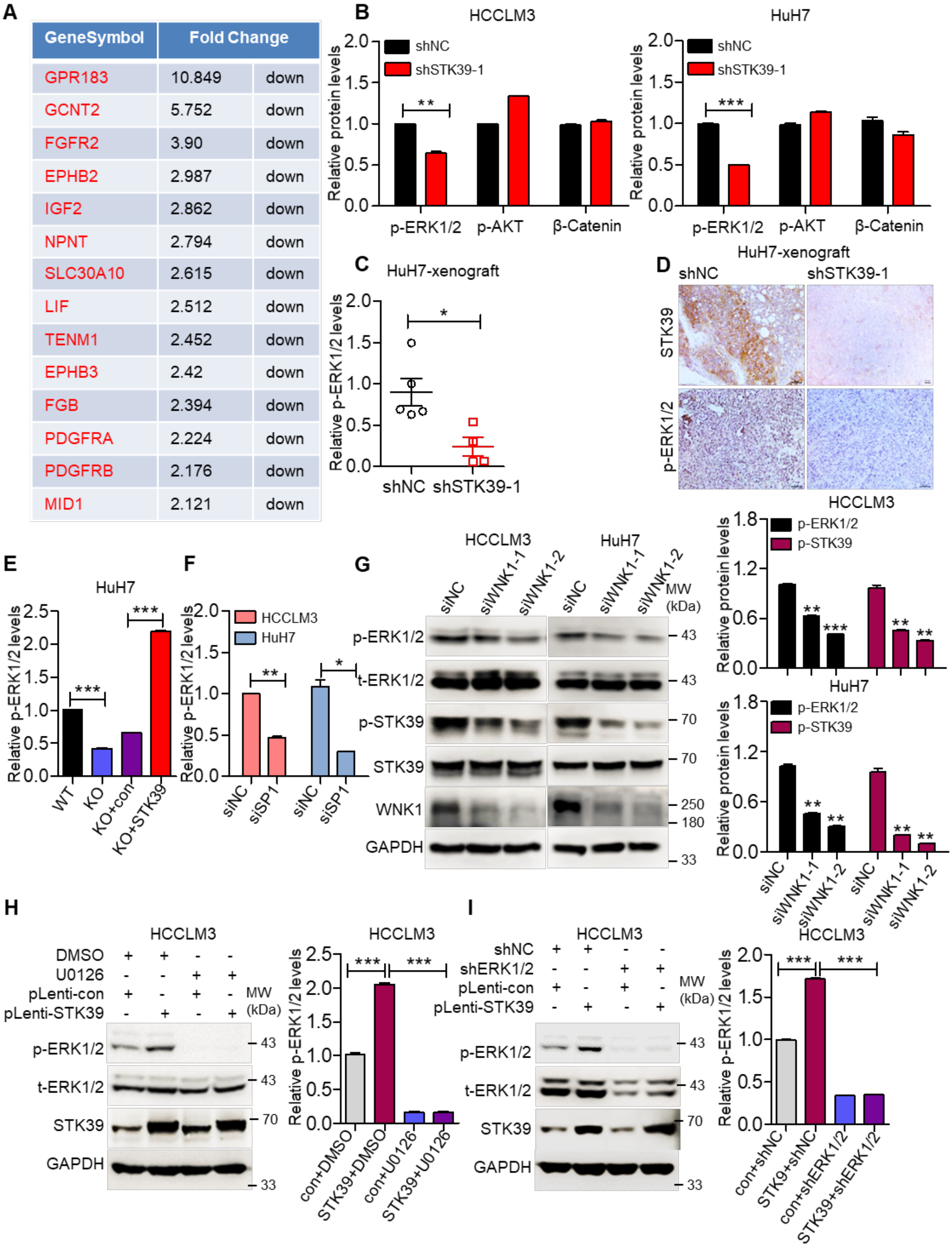

Figure S3. STK39 activates the ERK1/2 pathway in HCC cells, related to Figure 5. (A) The genes whose expression down-regulated more than 2-fold and enriched on the ERK1/2 signaling pathway; (B) The density of p-ERK1/2, p-AKT and β-Catenin in Figure 5C were assessed by Image J software; (C) The density of p-ERK1/2 in Figure 5D was measured by Image J software; (D) IHC analysis of the levels of

STK39 and p-ERK1/2 in tumor xenografts generated from subcutaneous injection of STK39 stable knockdown cells in nude mice; (E) The density of p-ERK1/2 in Figure 5E was measured by Image J software; (F) The density of p-ERK1/2 in Figure 5F was assessed by Image J software; (G) HCC cells were transfected with negative control siRNA or WNK1-specific siRNA for 72 h to knockdown WNK1, the levels of p-ERK, p-STK39 and WNK1 were examined by immunoblotting; (H) STK39-overexpression and control HCCLM3 cells were treated with or without U0126, the levels of p-ERK1/2 and STK39 were examined by immunoblotting; (I) ERK1/2-knockdown and control HCCLM3 were infected with control or STK39 overexpression lentivirus, the levels of p-ERK1/2 and STK39 were examined by immunoblotting. Data are shown as mean ±SEM. *p<0.05; **p<0.01; ***p<0.001.

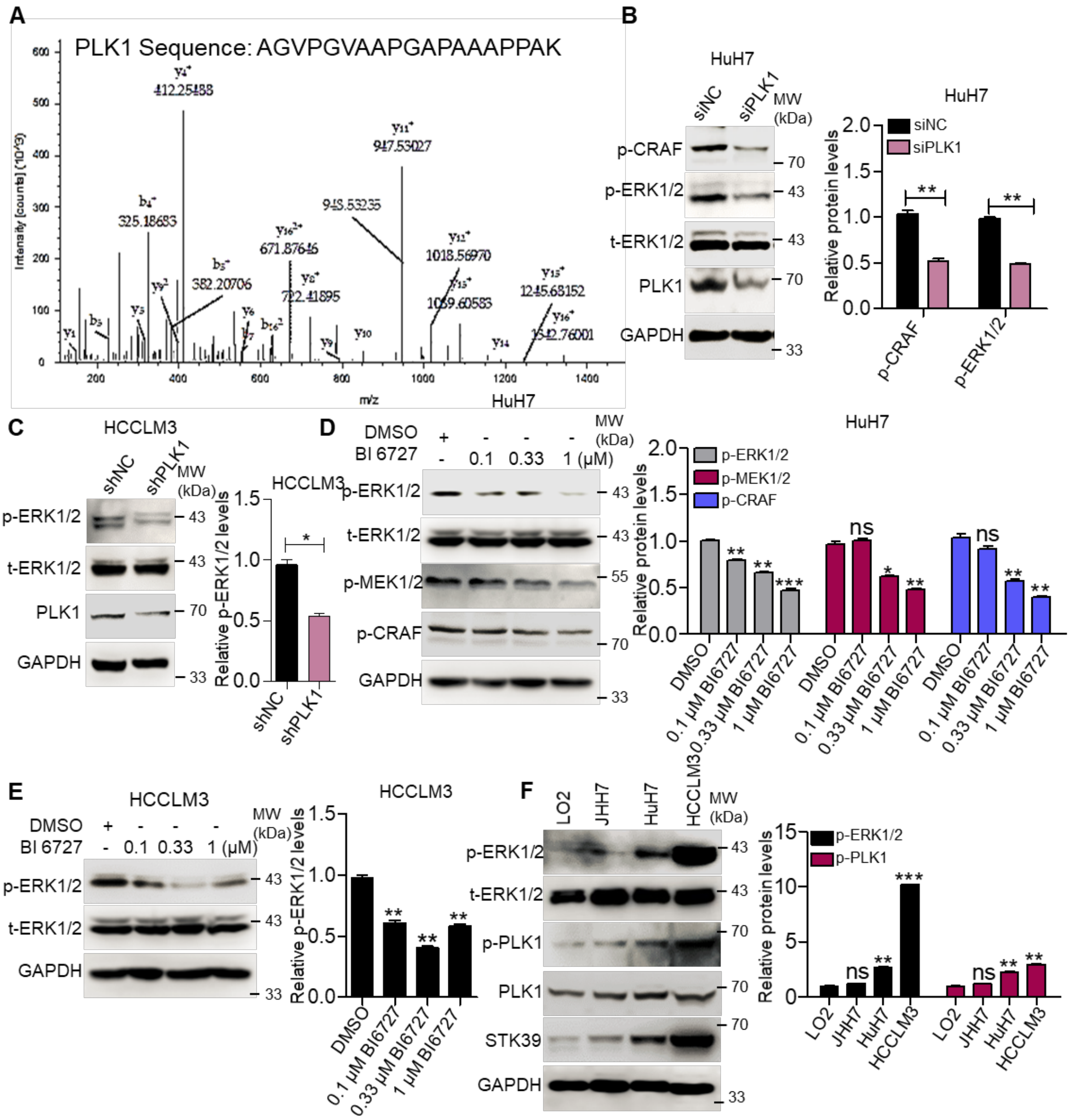

Figure S4. PLK1 activates ERK1/2 signaling pathway, related to Figure 6. (A) Mass spectrometry assay revealed that PLK1 is a partner of STK39; (B) Knockdown of PLK1 in HuH7 cells by siRNA, the levels of p-CRAF, p-ERK and PLK1 were assessed by immunoblotting; (C) Stable knockdown of PLK1 in HCCLM3 cells by shRNA, the levels of p-ERK1/2 and PLK1 were examined by immunoblotting; (D) HuH7 cells were treated with or without indicated concentrations of PLK1 inhibitor BI 6727, the levels of p-ERK1/2, p-MEK1/2 and p-CRAF were examined by immunoblotting; (E) HCCLM3 cells were treated with or without indicated concentrations of PLK1 inhibitor BI 6727, the level of p-ERK1/2 was examined by immunoblotting; (F) The levels of p-ERK, p-PLK1 and STK39 in LO2, JHH7, HuH7 and HCCLM3 cells were assessed by immunoblotting. Data are shown as mean ±SEM. *p<0.05; **p<0.01; ***p<0.001; ns, not significant.

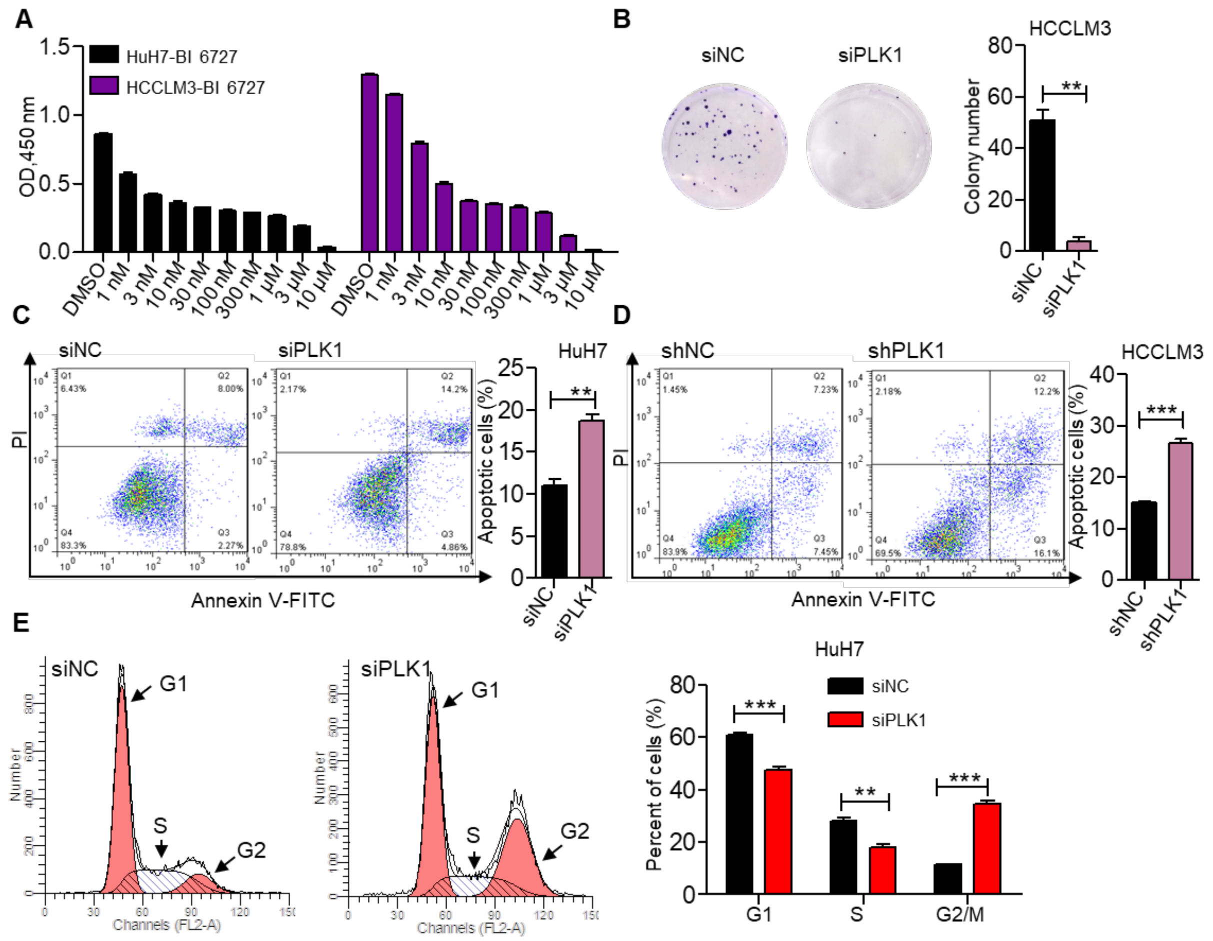

Figure S5. Inhibition or knockdown of PLK1 suppresses the growth, induces apoptosis and cell cycle arrest in HCC cells, related to Figure 7. (A) HCC cells were treated with indicated concentrations of PLK1 inhibitor BI 6727 for 3 days, the viability of cells was measured by CCK8 assay; (B) HCCLM3 cells were transfected with negative control siRNA or PLK1-specific siRNA for 48 h, the growth of cells was measured by colony formation assay; (C) Knockdown of PLK1 in HuH7 cells by siRNA, the percentage of apoptotic cells was analyzed by Annexin V-FITC/PI staining assay; (D) Knockdown of PLK1 in HCCLM3 cells by shRNA, the percentage of apoptotic cells was analyzed by Annexin V-FITC/PI staining assay; (E) Knockdown of PLK1 in HuH7 cells by siRNA, cell cycle was analyzed by flow cytometry. Data are shown as mean ±SEM.**p<0.01; ***p<0.001.
